# Selective Area Band Engineering of Graphene using Cobalt-Mediated Oxidation

**DOI:** 10.1038/srep15380

**Published:** 2015-10-21

**Authors:** Paul F. Bazylewski, Van Luan Nguyen, Robert P.C. Bauer, Adrian H. Hunt, Eamon J. G. McDermott, Brett D. Leedahl, Andrey I. Kukharenko, Seif O. Cholakh, Ernst Z. Kurmaev, Peter Blaha, Alexander Moewes, Young Hee Lee, Gap Soo Chang

**Affiliations:** 1 Department of Physics and Engineering Physics, University of Saskatchewan, 116 Science Place, Saskatoon, SK, S7N 5E2, Canada; 2 Center for Integrated Nanostructure Physics, Institute for Basic Science, Sungkyunkwan University, Suwon 440-746, Korea; 3 Institute Materials Chemistry, Vienna University of Technology, Getreidemarkt 9/165-TC, A-1060 Vienna, Austria; 4 Institute of Metal Physics, Russian Academy of Sciences-Ural Division, 620990 Yekaterinburg, Russia; 5 Ural Federal University, 19 Mira Str., 620002 Yekaterinburg, Russia; 6 Department of Physics and Department of Energy Science, Sungkyunkwan University, Suwon 440-746, Korea

## Abstract

This study reports a scalable and economical method to open a band gap in single layer graphene by deposition of cobalt metal on its surface using physical vapor deposition in high vacuum. At low cobalt thickness, clusters form at impurity sites on the graphene without etching or damaging the graphene. When exposed to oxygen at room temperature, oxygen functional groups form in proportion to the cobalt thickness that modify the graphene band structure. Cobalt/Graphene resulting from this treatment can support a band gap of 0.30 eV, while remaining largely undamaged to preserve its structural and electrical properties. A mechanism of cobalt-mediated band opening is proposed as a two-step process starting with charge transfer from metal to graphene, followed by formation of oxides where cobalt has been deposited. Contributions from the formation of both CoO and oxygen functional groups on graphene affect the electronic structure to open a band gap. This study demonstrates that cobalt-mediated oxidation is a viable method to introduce a band gap into graphene at room temperature that could be applicable in electronics applications.

There has been much recent progress in the development of graphene in electronics for a wide range of device applications including field-effect transistors (FETs)^1^,^2^, photovoltaic cells^3^,^4^, sensors^5^, and spintronic FETs^6^. Graphene is attractive for many applications due to its unique properties including high charge carrier mobility and thermal conductivity, coupled with a large surface area for catalysis or sensing applications. However, utilizing graphene effectively in scalable semiconductor technologies has been limited by difficulties in engineering it with a reproducible and tunable band gap in the selected area. Tailoring graphene’s electronic properties, in particular the electronic band gap, is critical for graphene-based devices to be brought to their fullest potential. Several studies have resulted in successful methods to open a band gap in graphene. These include the use of multilayer graphene functionalized with organic molecules in order to break the layer symmetry^7^,^8^,^9^,^10^, heterogeneous or single atom substitutions^11^,^12^, quantum confinement using graphene nano-ribbons^13^,^14^, and oxygen functionalization related to graphene oxide (GO)^15^,^16^,^17^,^18^,^19^,^20^. Using bilayer graphene, a band gap may be opened by charge transfer doping from either adsorbed molecules or a substrate to open a tunable gap of 50–100 meV, as shown by Park *et al.* where bilayer graphene was used in single gate *n*-type FETs^1^. Nano-ribbons utilizing the quantum confinement effect can also achieve a reproducible, controllable band gap, where ribbons were fabricated from molecular precursors obtaining a gap of 1.4 or 2.5 eV^14^. Nano-ribbons have been integrated into devices using metal-nanowire-etching to fabricate a ribbon-based device *in situ*, but at a considerable cost in fabrication complexity^19^. The most prolific example of chemical functionalization approach is GO where formation of *sp*^3^ and *sp*^2^ hybridizations due to oxygen bonding can open a band gap of 0.02 to 3.5 eV, depending on the number of graphene layers and fabrication process (reduction level)^20^. With implementation of GO in devices, the concerns become reliable fabrication of GO with reproducible properties in a scalable manner and the risk of introducing defects and fragmenting the GO by thermal annealing. Despite impressive results achieved, a method to induce a reproducible band gap in the selected area of graphene with less complexity remains elusive.

This study reports the use of graphene modified with cobalt to induce an electronic band gap in a manner that is scalable and low in complexity. Other studies have examined Co on graphene as either adatoms or layers using experimental^21^ and theoretical^22^ means, but have not to date revealed a band gap in graphene using this method. Single layer graphene used in this study was synthesized on Cu foil at the Center for Integrated Nanostructure Physics (Sungkyunkwan University, Korea). To prevent interaction with the Cu substrate that could shift the graphene Fermi level, graphene was transferred to SiO_2_:Si substrates using a well-known polymethylmethacrylate transfer method^23^. Graphene samples were decorated with a Co using physical vapor deposition (PVD) by resistive heating of a tungsten boat. At a low deposition rate, PVD produces a very low nucleation density on the graphene surface, resulting in slow heterogeneous cluster growth. Afterwards samples were exposed to oxygen at atmospheric pressure, to form both carbon and cobalt oxides. At a cobalt thickness of 0.12 nm on graphene/SiO_2_ a band gap forms at room temperature due to formation of both oxygen functional groups on the graphene and CoO.

## Results and Discussion

**Figure 1:**
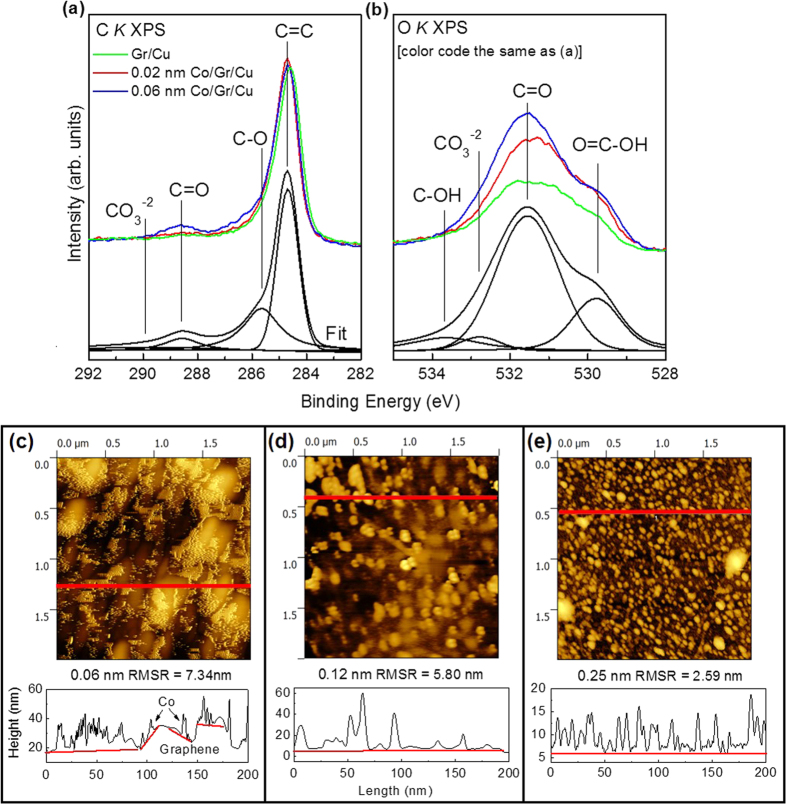
Photoelectron and Raman Spectroscopy of graphene samples on Cu and SiO_2_ substrates. (**a**) C *K* XPS, (**b**) O *K* XPS of Co/Graphene/Cu with peaks fitted using Voigt functions to locate the component peaks of the 0.06 nm Co/graphene spectrum. AFM results of a 2 × 2 μm^2^ area topography for Co/Graphene/SiO_2_ with (**c**) 0.06 nm, (**d**) 0.12 nm, and (**e**) 0.25 nm of Co with roughness profiles below. The roughness profile shown corresponds to the red line in the upper panel images.

Figure 1(a,b) detail X-ray photoelectron spectroscopy (XPS) measurements at the carbon and oxygen *K*-edges of Co/graphene/Cu samples to examine the carbon and oxygen bonding environments. For Co-deposited graphene, the C = O and C−O bonds are formed and increase with Co thickness. Notably some native oxide groups (C = O, C−O) are present in low concentration on as prepared graphene/Cu, possibly due to adsorbed water on the graphene surface after exposure to ambient atmosphere. In the O K XPS there may also be a contribution from cobalt oxides in peaks in the range of 529–533 eV, but they are too small in intensity to be differentiated unambiguously using peak fitting^24^. To probe formation of oxides, cobalt bonding is examined more closely with X-ray absorption spectroscopy.

The graphene surface was imaged after Co deposition using atomic force microscopy (AFM), with images of Co clusters shown in Fig. 1(c–e). The Co exhibits Volmer-Weber growth on the graphene surface where islands are formed at nucleation sites preferentially without homogeneous growth across the surface. This growth type is preferred when adatom–adatom interactions are stronger than those of the adatom with the surface (which is the case for graphene without defects), leading to the formation of clusters or islands. At 0.06 nm Co, the clusters are small in diameter and aggregate in local areas of 2–3 μm^2^. At 0.12 nm thickness, the clusters become substantially larger in diameter but are spaced further apart, and do not homogeneously cover the graphene surface. This growth pattern supports nucleation at hydrocarbon or impurity sites, limiting vacancy formation. At 0.25 nm, the Co covers the surface more uniformly, presenting as many smaller clusters spaced closely together. When Co is deposited, charge transfer makes the graphene surface more energetically favorable for uniform wetting, resulting in more homogeneous coverage. This is reflected in the root-mean-square roughness showing the lowest value across all samples for 0.25 nm. Note that some local wrinkling of the graphene is evident in the roughness profile shown in Fig. 1(c) where red lines are used to mark the boundary between the graphene and Co clusters.

**Figure 2:**
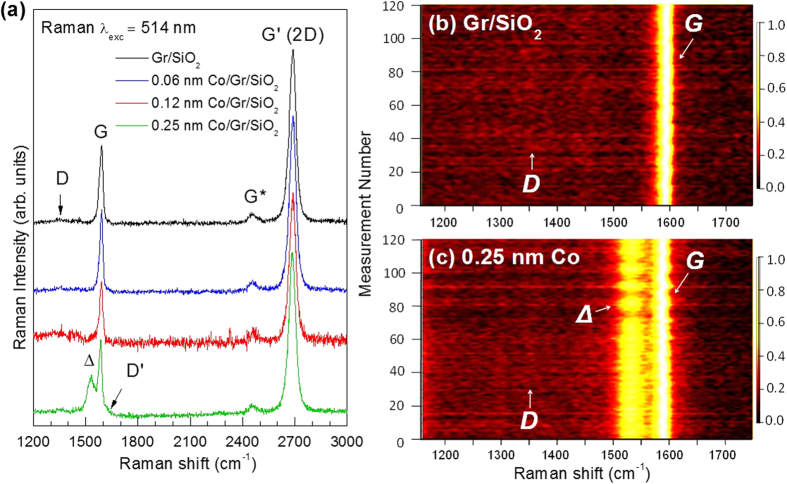
(**a**) Raman spectra of the Co/Graphene/SiO_2_ compared to un-deposited Graphene/SiO_2_. (**b**) and (**c**) show Raman mapping results of the *D* and *G*-bands for representative samples of pristine graphene/SiO_2_ and Co(0.25 nm)/Graphene, respectively.

We verified that the graphene was undamaged by solvents used in the transfer process using Raman spectroscopy measurements before and after Co deposition [Fig. 2(a)]. After the transfer, the graphene remains intact with clearly defined *G* and *G’* (*2D*) bands. Below 0.25 nm thickness of Co, the spectra appear very similar to undeposited graphene/SiO_2_ while a red shift is observed in both *G* (6 cm^−1^) and *G’* (3.8 cm^−1^) bands at 0.25 nm of Co. This small shift of the *G*-band to lower wavenumber is attributed to charge transfer from the Co to the graphene^23^,^24^,^25^. A shift due to strain is unlikely because a red shift would indicate reduced strain after Co deposition. At 0.12 nm of Co thickness, broadening of the *G*-band is visible that develops into a shoulder peak at 1531 cm^−1^ for 0.25 nm Co. The similar *G*-band broadening has been reported for Cr-deposited on graphene^26^, and in semiconducting single-walled carbon nanotubes (S-SWNT)^27^. In S-SWNTs this band has a Lorentzian lineshape and arises from vibrations in carbon atoms along the circumferential direction of the tube. Since only a suppressed D-band is visible, a high level of graphene curvature similar to a CNT is unlikely. In this case this feature arises from a removal of degeneracy in the G-band stretching mode due to Co deposition. This feature is resonantly enhanced in intensity due to additional electrons transferred from the Co, causing an increase in electron-phonon interaction. The ratio of integrated peak intensity, *A*(*G’*)/*A*(*G*), is calculated to be ~4 or greater for all cases (Table 1), showing the graphene is indeed a single layer and has not become folded or heavily wrinkled from the transfer process^27^,^28^. In addition, a very low *A*(*D*)/*A*(*G*) ratio (below 0.1) indicates nearly defect-free graphene. To verify that defects were not present over a larger area of the graphene surface, Raman mapping was performed with 120 measurements in a 10 um^2^ area [Fig. 2(b,c)]. A very low *D*-band intensity and consistent *G*-band signal is observed for graphene transferred SiO_2_, which remains consistent for Co ≤ 0.25 nm deposited on the surface. The Δ-band is also observed to be continuous in intensity for the majority of the measurements in Fig. 2(c), indicating uniform and homogeneous Co coverage in agreement with AFM results.

With minimal defects in the graphene, damage induced by Co or Co oxides can be ruled out as a possible cause of band gap opening. This result is notable because Co and other transition metals can produce vacancy-related defects in graphene. The use of metal atoms to etch or cleave graphene in a controlled way is a well-known procedure using many metals including Ni, Cr, Pd, Al, or Ti in conjunction with oxidation or hydrogenation at elevated temperatures^29^. Although we did not anneal the samples, Boukhvalov *et al.* have predicted that metal adatoms (Fe, Co, Ni, or Al) lower the vacancy formation energy in free-standing graphene without assistance from heat^30^. In particular, Co is shown to have the lowest energy for vacancy and bi-vacancy formation compared to other metals when deposited on graphene. However, the vacancy formation in that study was simulated for unsupported pristine graphene, which is not our case. To understand the metal behavior, we consider several experimental studies using atomic resolution topography images showing that regardless of the fabrication method used, the graphene surface contains adsorbed hydrocarbon groups that are randomly distributed^31^,^32^. Studies on such systems examining metal cluster growth with high resolution transmission electron microscope (HR-TEM) report metal adatoms deposited on the surface prefer to aggregate at hydrocarbon sites, rather than the pristine honeycomb graphene surface^31^. Therefore covalent bonding between graphene and metal adatoms with subsequent vacancy formation is limited. This is in agreement with the low *D*-band signal measured on our samples, indicating that Co does not induce vacancy formation and thus does not directly manipulate the graphene band structure.

**Figure 3:**
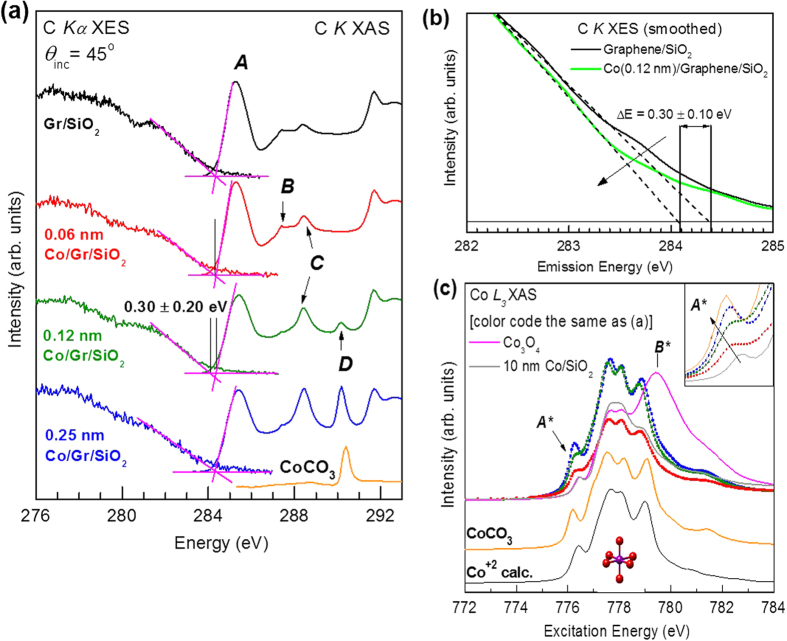
X-ray spectroscopy results of Co/graphene/SiO_2_. (**a**) Non-resonant C *K*α XES and C *K* XAS spectra, (**b**) smoothed XES of graphene/SiO_2_ compared to Graphene with 0.12 nm of Co deposited, and (**c**) Co *L*_*2,3*_ XAS spectra for Co/graphene/SiO_2_ samples compared to pristine graphene and references including a Co^2+^ simulation.

The graphene electronic band structure and a band gap induction were investigated using synchrotron-based X-ray absorption (XAS) and emission (XES) measurements. Figure 3(a) shows the details of C *K* XAS and C *Kα* XES which probe the partial density of unoccupied and occupied C 2*p* states, respectively. Over the sample set the unoccupied states exhibit several π* peaks that change in relative intensity with visible trends. The main C = C π* feature (*A*) appearing at 285.3 eV representing the graphene conduction band (CB) edge, is observed to shift slightly to higher energy. Close inspection reveals an observable shift of *A* to higher excitation energy for 0.06–0.12 nm of Co compared to undeposited graphene/SiO_2_. Literature works describing C *K* XAS measurements of graphene deposited on metallic films or decorated with metal clusters show a splitting of the 285.0 eV π* feature due to metal-graphene covalent bonding^33^,^34^. Furthermore, when metal-graphene bonding occurs the sharp C-C σ* excitonic peak at 292 eV is broadened by 1–2 eV and reduced in intensity^33^,^34^. It is clear that neither splitting of *A* nor suppression of the excitonic peak occurs in our samples, suggesting an absence of metal-graphene covalent bonding. Higher energy peaks are also evident that change in intensity and shape upon Co deposition, with some also present in undeposited graphene. Peaks *B* (287.4 eV) and *C* (288.3 eV) are due to the presence of hydroxyl (C-OH) or epoxide (C-O-C), and carbonyl (C =O) or carboxyl (COOH) functional groups, some of which may be remnant from the transfer process^35^,^36^. It is likely that peak *B* does not arise from bonding to functional groups, but rather from carbon sites that have been perturbed by nearby functional groups^35^. This detail is important as bonding of epoxide groups (which are in the energy position of peak *B*) could act to break up the graphene over time^36^. Peak *D* exhibiting small or negligible intensity until 0.25 nm Co is reached, is attributed to the carbonate (CO_3_) structure (290.2 eV) which is verified by comparison to CoCO_3_ (Alfa Aesar 99.95%) reference powder. Carbonates may only develop with greater Co thickness because formation of Co-C bonding is energetically unfavorable until reaching a threshold amount of Co. Peak *C* has a more obvious trend of intensity increase proportional to Co thickness that is consistent with XPS measurements showing a fractional increase of C = O/C-O bonds upon Co deposition. Uniquely in our samples, oxide formation appears to be mediated by the presence of Co.

To quantitatively determine the band gap energy, the leading edges of C *K* XAS and the corresponding C *K*α XES spectrum were extrapolated to the baseline by fitting a linear line to the edge of each spectra to determine the conduction-band-minimum (E_CBM_) and valence-band-maximum (E_VBM_), respectively^37^. Using this treatment, the E_CBM_ and E_VBM_ of undeposited graphene/SiO_2_ intersect at 284.4 eV, indicating a band gap of 0 eV. After depositing 0.12 nm of Co, a band gap of 0.30 eV ± 0.14 eV opens. To our knowledge this is the first reported case of band gap opening in graphene as a result of metal deposition on its surface. The error of 0.14 eV is the maximum expected one when calculating an absolute band gap in this case. Using this technique maximum errors in the range of 0.1–0.2 eV are typical depending on the energy range of the measurements^38^,^39^. This error arises due to core-hole effects in the XAS combined with the resolving power of the beamlines used, which is approximately 0.04 eV in this energy range. The shift of the occupied states is visualized in Fig. 3(b) showing XES of pristine graphene compared to graphene with 0.12 nm of Co deposited. The suppression of spectral intensity near the band edge and relative shift between XES spectra is clear with a shift of the density of states (DOS) to lower energy. Note that in this case the estimated error is less for spectra taken immediately after one another under the same conditions, and for XES are also not complicated by the core-hole shift that appears in XAS.

Note that the band gap opening occurs when the concentration of carbonate (peak *D*) is low and that of the other oxide species is comparatively high (peak *C*). At Co thickness of 0.25 nm, no band gap is observed similar to undeposited graphene. 0.25 nm of Co is approximately a monolayer; at this thickness enough Co is present to revert the Co/graphene composite to a metallic state. It would seem that the amount of Co present, as well as how it bonds to the graphene lattice, has a strong influence on the degree of functionalization and hence the band gap. A band gap of 0.05–0.30 eV for Co concentration below 0.25 nm can be rationalized by considering graphene oxide and the O:C ratio. A gap of this size has been predicted to open at an O:C ratio of 27.8–50% in rGO^15^. Experimentally a gap of 0.45 eV has been demonstrated under 28% O:C ratio for oxidized monolayer graphene^18^. This is in qualitative agreement with the Co-graphene system when considering the relative intensities of peaks *C* and *D* compared to peak *A* at 0.12 nm Co/Graphene/SiO_2_.

The other main component not yet considered is the cobalt itself. To probe the bonding environment of Co sites, Co *L*_*2,3*_ XAS was measured [Fig. 3(c)]. Two peak positions are labeled, A* (776.2 eV) representing the onset of Co unoccupied states, and B* (779.4 eV), a characteristic peak of Co^3^ in Co_3_O_4_. At first glance, Co/graphene/SiO_2_ shows sharper multiplet features associated with metal-oxides when compared to a 10 nm metallic reference Co film, and does not present a visible peak B*. To confirm a divalent Co (Co^2+^) oxidation state, multi-ligand field theory (MLFT) simulations were performed and fit to the 0.25 nm Co result, identifying Co^2+^ in nearly octahedral (C_4h_) symmetry, consistent with high spin CoO. Shown enlarged in the inset of Fig. 3(c), A* shifts to lower energy by 0.15 eV due to charge transfer caused by a transition from CoO to CoCO_3_. This shift is identified by comparison to reference CoCO_3_ powder; the lineshape closely matches that of 0.25 nm Co and reproduces the shift in A*.

**Figure 4:**
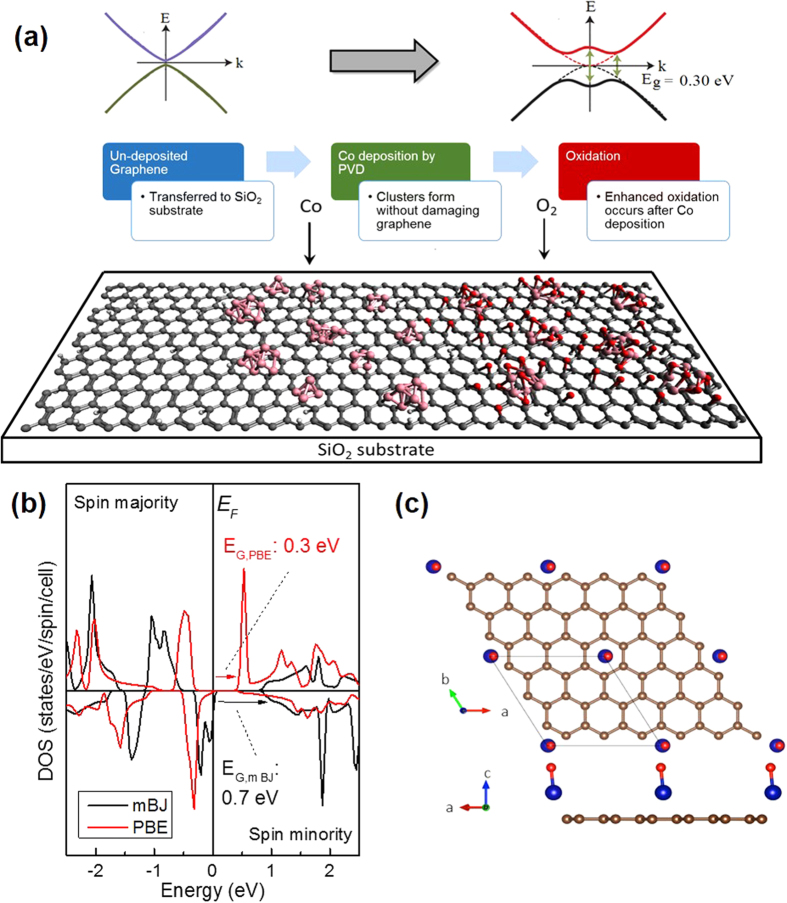
(**a**) Schematic representation of band opening in Co/graphene/SiO_2_. Co deposition on graphene followed by local graphene oxidation and Co-oxide formation. This procedure creates semiconducting graphene that may be adjacent to un-deposited conducting graphene. (**b**) Simulated density of states for CoO/Graphene using mBJ and PBE force relaxation methods. A semiconducting gap is predicted in both spin channels for both methods when CoO is present as a monomer. (**c**) Image of CoO/graphene used for simulations.

With the results presented, we can speculate that the mechanism of band opening is related to Co-mediated oxidation of the graphene [Fig. 4(a)]. For single layer graphene, charge transfer alone from metallic impurities does not open a semiconducting gap, but rather shifts the Fermi level according to the doping type^40^. In our Co/graphene system, a two-step process takes place beginning with charge transfer to the graphene, followed by oxidation that opens a band gap. When Co is in close proximity (3 Å) to graphene, minor charge doping of the graphene can occur^22^. This results in a higher electron charge on the graphene locally, and greater affinity for covalent bonding with oxygen. As observed in the XAS, oxygen functional groups form more readily in the presence of Co, but are also mediated by it to a fixed concentration. A band gap is then opened due to *sp*^3^ bond formation when a high enough oxygen concentration is reached in the form of carbonyl or hydroxyl groups.

To further examine this system, electronic structure simulations have been used to examine the effect of CoO on the graphene band structure. Calculations were focused on CoO because a band gap is observed only when the concentration of carbonates is low compared to CoO and other oxygen functional groups. This is possibly because formation of a carbonate requires a site at a vacancy, defect or edge, meaning that carbonates initially formed are anchored at defect sites natively present on undeposited graphene. This is supported by the Raman measurements which do not show evidence of graphene etching after metal deposition. From Fig. 3(a), the only significant changes to the C *K* XAS spectral shape between 0.12 nm and 0.25 nm are intensity increases in peaks *C* and *D*. This suggests that CoCO_3_ contributes to closing a band gap that is induced by other factors, and therefore calculations were focused on isolating the effect of CoO.

Simulations of a low-coverage CoO/graphene system have been performed using the all-electron WIEN2k code [Fig. 4(c)]^41^. Notably after force relaxation the underlying graphene sheet does not buckle or otherwise become deformed, even if DFT forces, which neglect van der Waals interactions when using local density approximation or generalized gradient approximation (GGA) functionals, are corrected for dispersion errors using the DFT-D3 code. CoO molecules were found to preferentially occupy the hollow sites above the hexagonally symmetric vacancies in graphene rings, as supported by previous studies^42^,^43^. Figure 4(b) shows the results of a total DOS simulation for a CoO decorated graphene sheet. Upon introduction of CoO and force relaxation using the Perdew–Burke–Ernzerhof (PBE) functional, a semiconducting gap develops in spin channels for both the total and carbon 2*p* partial DOS. Similar calculations using GGA plus van der Waals correction with adsorbed CoO exhibit a band gap opening in the total DOS (including the carbon 2*p* projection) above a Co surface loading of 11% (e.g. in the case of one CoO isolated on a 3 × 3 supercell of the graphene unit cell). At lower CoO surface loading the graphene layer remains metallic. Since GGA in the PBE formulation is widely known to underestimate band gaps in materials, we have also used the Tran-Blaha modified Becke-Johnson (mBJ) potential as a correction, resulting in the band gap of 0.3 eV increasing to 0.7 eV^44^. The gap is opened by hybridization of Co 3*d*4*s* states with graphene 2*p* states. Electrons in hybridized orbitals are then partially localized by the Co-O bond. This localization effectively makes these electrons unavailable for conduction, resulting in partially filled states near the valence band edge and a band gap opening. Although this system was not developed experimentally, it shows that only CoO may be required to open a band gap in graphene.

From the results and discussion above, we have developed a low complexity scalable method to open a band gap in graphene of up to 0.30 eV. We propose a plausible mechanism of band gap opening as a two step-process of minor charge transfer to the graphene from Co, followed by formation oxide groups including CoO in a concentration mediated by the Co. The use of PVD with a very slow deposition rate and low cobalt thickness allowed for metal clusters to grow at impurity sites without etching the graphene. Exposure to oxygen forms hydroxyl and carbonyl groups on the graphene where Co has been deposited, which can lead to sufficient O:C ratio to open a semiconducting band gap. This method is unique in that *sp*^3^ bond formation occurs to open a band gap, but only in the local area where Co is deposited, in low enough concentration to prevent deformation or damage of the graphene. Damage and fragmentation of graphene are a weakness of other fabrication methods that rely on solution-based methods with thermal heating to functionalize graphene in a controllable way. Theoretical calculations also reveal an additional contribution from CoO that can result in a band gap opening up to 0.7 eV due to CoO alone. These results point to a dual effect of minor oxidation of the graphene combined with CoO formation that act together to open a band gap.

Co/graphene may be attractive for use in patterned electronic devices by using masking to deposit on a selected area of a graphene substrate. This method is therefore scalable to the maximum size of graphene sheets that can be obtained by conventional growth techniques. Most importantly, the band gap produced is of sufficient size to be of use in semiconducting applications, and is stable at room temperature under exposure to ambient conditions. These results suggest that cobalt-mediated oxidation is a viable method to introduce a band gap into graphene that could be feasible in other research areas requiring controlled oxidation of organic films.

## Methods

### Preparation of Cobalt on Graphene

Graphene was grown on polished Cu foil (100 μm thickness) using atmospheric pressure chemical vapor deposition^45^,^46^, and transferred to SiO_2_ by a standard chemical procedure (refer to ref. 25 for solution transfer details). Co metal was deposited onto graphene/SiO_2_ using physical vapor deposition (PVD) with Co metal powder (Sigma Aldrich 99.995% metals basis) as the source material in a tungsten boat. A deposition rate of 0.02 Å/s was achieved using a crystal thickness monitor *in situ*. The substrates were held at room temperature prior to deposition, and allowed to heat as cobalt was deposited. During deposition the pressure did not go above 10^−5^ torr. Reported final thicknesses are as recorded from the thickness monitor. Afterwards samples were exposed to oxygen at atmospheric pressure, to form both carbon and cobalt oxides.

### Spectroscopic Characterization

XAS in total electron yield mode and XES measurements were performed at the Resonant Inelastic X-ray Scattering beam line at the Canadian Light Source of the University of Saskatchewan and at Beamline 8.0.1 of the Advanced Light Source in Berkeley, CA. All spectra were measured at a 45° angle of incidence. XAS spectra were measured in total electron yield mode and normalized to the incoming photon flux as recorded by Au mesh and intensity normalized to a constant background as follows: C *1*s XAS at 310 eV, C *K*α XES at 265 eV, and Co *L*_2,3_ XAS at 810 eV. Raman Spectroscopy measurements were performed using a Renishaw inVia Raman Microscope with a 514 nm laser source at a power of 0.01 W and 1800 lines/mm grating. XPS Core-level and valence-band spectra measurements were made using PHI XPS Versaprobe 5000 spectrometer (ULVAC-Physical Electronics, USA) at the Federal Ural University Lab in Yekaterinburg, Russia. The XPS spectra were recorded using Al Kα (1486.6 eV) 100 μm spot mode with X-ray power load on the sample less than 25 W and pressure of 10^−7^ Pa. The spectra were processed using ULVAC-PHI MultiPak Software 9.3 and the residual background was removed with the Tougard method.

### Theoretical Calculations

For the CoO/graphene structure, DFT simulations were performed using the WIEN2k code by placing a single Co on 3 × 3, 4 × 4 and 5 × 5 supercells of graphene (18, 32 and 50 carbon atoms, respectively). The graphene sheets were isolated from each other by 20 angstroms vacuum. The position of Co was relaxed until all forces were below 1 mRy/a.u. Similar relaxation process was done when an O was added above the Co. A gamma-point centered mesh of 20 points was sampled in *k*-space, with a plane wave expansion of up to RK max of 5.0, and an convergence of 0.1 mRy was achieved. Muffin tin sphere sizes were C:1.28, Co:1.49, O: 1.28. The PBE functional^47^ as well as the modified Becke-Johnson potential^40^ were used. Dispersion forces were corrected in the force relaxation through the use of the DFT-D3 code^41^. MLFT simulations used a single impurity Anderson model, including multiplet effects, crystal field splittings, and hybridization with ligands. The simulations employ a model Hamiltonian approach where adjustable parameters are fit to the experiment. These parameters include crystal field splitting parameters (10Dq, D*σ*, D*τ*), symmetry-dependent hopping integrals (*V*_*e*_*, V*_*t2*_), charge transfer energy (Δ), the difference between the onsite Coulomb repulsion and the core hole potential (*U−Q*), and the ligand bandwidth (*W*) and shape^48^,^49^.

## Additional Information

**How to cite this article**: Bazylewski, P. F. *et al.* Selective Area Band Engineering of Graphene using Cobalt-Mediated Oxidation. *Sci. Rep.* **5**, 15380; doi: 10.1038/srep15380 (2015).

## Figures and Tables

**Table 1:** Raman band locations and integrated peak area ratios.

**Sample**	**Peak locations (cm**^**−1**^)						**Integrated Peak Area ratios**		
	***D***	**∆**	***G***	***D’***	G*	***G’***	**A(*****D*****)/A(*****G’***)	**A(*****D*****)/A(*****G***)	**A(*****G’*****)/A(*****G***)
Gr/SiO_2_	1353	*NA*	1593	*NA*	2455	2689	0.00325	0.0138	4.26
Co 0.06 nm	1353	*NA*	1593	*NA*	2458	2689	0.00108	0.0479	4.44
Co 0.12 nm	1353	*NA*	1589	*NA*	2459	2688	0.0628	0.2504	3.98
Co 0.25 nm	1353	1531	1586	1634	2458	2686	0.0109	0.0724	6.71
